# Urinary Interleukin-8 Is a Biomarker of Stress in Emergency Physicians, Especially with Advancing Age — The JOBSTRESS* Randomized Trial

**DOI:** 10.1371/journal.pone.0071658

**Published:** 2013-08-19

**Authors:** Frédéric Dutheil, Marion Trousselard, Christophe Perrier, Gérard Lac, Alain Chamoux, Martine Duclos, Geraldine Naughton, George Mnatzaganian, Jeannot Schmidt

**Affiliations:** 1 Emergency Department, University Hospital (CHU), G. Montpied Hospital, Clermont-Ferrand, France; 2 Department of Occupational Medicine, University Hospital (CHU), G. Montpied Hospital, Clermont-Ferrand, France; 3 Laboratory of Metabolic Adaptations to Exercise in Physiological and Pathological Conditions EA3533, Blaise Pascal University, Clermont-Ferrand, France; 4 School of Exercise Science, Australian Catholic University, Melbourne, Victoria, Australia; 5 Department of Sport Medicine and Functional Exploration, University Hospital (CHU), G. Montpied Hospital, Clermont-Ferrand, France; 6 Laboratory of Stress Neurophysiology, IRBA, Brétigny sur Orge, France; 7 INRA, UMR 1019, UNH, CRNH Auvergne, Clermont-Ferrand, France; 8 Faculty of Health Sciences, Australian Catholic University, East Melbourne, Victoria, Australia; University of Rochester, United States of America

## Abstract

**Background:**

Emergency physicians are exposed to greater stress during a 24-hour shift (24 hS) than a 14-hour night shift (14 hS), with an impact lasting several days. Interleukin-8 (IL-8) is postulated to be a chronic stress biomarker. However, no studies have tracked IL-8 over several shifts or used it for monitoring short-term residual stress. The IL-8 response to the shifts may also increase with age. Conveniently, IL-8 can be measured non-intrusively from urine.

**Methods:**

We conducted a shifts-randomized trial comparing 17 emergency physicians’ urinary IL-8 levels during a 24 hS, a 14 hS, and a control day (clerical work on return from leave). Mean levels of IL-8 were compared using a Wilcoxon matched-pairs test. Independent associations of key factors including shifts, stress, and age with IL-8 levels were further assessed in a multivariable generalized estimating equations model.

**Results:**

Mean urinary IL-8 levels almost doubled during and after a 24 hS compared with a 14 hS or a control day. Furthermore, IL-8 levels failed to return to control values at the end of the third day after the shift despite a rest day following the 24 hS. In the multivariable model, engaging in a 24 hS, self-reported stress, and age were independently associated with higher IL-8 levels. A 24 hS significantly increased IL-8 levels by 1.9 ng (p = .007). Similarly, for every unit increase in self-reported stress, there was a 0.11 ng increase in IL-8 levels (p = .003); and for every one year advance in age of physicians, IL-8 levels also increased by 0.11 ng (p = .018).

**Conclusion:**

The 24 hS generated a prolonged response of the immune system. Urinary IL-8 was a strong biomarker of stress under intensive and prolonged demands, both acutely and over time. Because elevated IL-8 levels are associated with cardiovascular disease and negative psychological consequences, we suggest that emergency physicians limit their exposure to 24 hS, especially with advancing age.

## Introduction

Emergency medicine is a unique specialty focusing on a breadth of acute care, on demand [1], [2]. Shift work is also a fundamental component of emergency medicine, and is associated with chronic stress, including stress at work. Consequently, stress may lead to symptoms of mental exhaustion, physical fatigue, detachment from work, and feelings of diminished competence [3]. Emergency physicians (EPs) are exposed to a complex interplay between stress (life-and-death emergencies – a defining characteristic of their job), sleep deprivation, and fatigue due to repeated changes in, and duration of shifts [4]. Work-related exhaustion can lead to various physical and psychological symptoms, and also may be associated with delayed decision-making [5]. The combined effects of stress and fatigue can impact on job performance, often resulting in otherwise preventable medical errors [6]. Moreover, prolonged stress may expose EPs to a higher risk of multiple diseases [7], [8], [9], [10], predominantly systemic inflammation [11] and coronary heart disease [12]. All these contribute to the premature departure of EPs to other specialties [13].

Among the multiple cytokines involved in well being and depressive symptoms [11], [14], [15], [16], interleukin-8 (IL-8) is also linked with inflammatory responses [11] and the pathogenesis of coronary heart disease and atherosclerosis [11], [17], [18], [19], [20]. Further, IL-8 was postulated to be a chronic stress biomarker in shift work nurses from an acute care department [21]. Although the prolonged immune response of IL-8 to stress has not been studied, some cytokines have been reported to require as many as five days to return to basal levels following a stressful event [22]. Similar to most of the cytokines, IL-8 is mainly produced by macrophages [23]. With advancing age, macrophages remain activated for longer and respond to multiple stimuli [23]. In addition, IL-8 has recently shown links to high physical demands [24]. Lastly, studies showing links between sleep and immune responses consistently involve artificially induced sleep deprivation protocols [25], [26], [27], [28]. However, no studies have tracked IL-8 over several shifts nor explored its potential for monitoring short-term residual stress, and possible links with advancing age. Furthermore, no trial has assessed the role of IL-8 in a natural model of sleep deprivation induced by work. Conveniently, IL-8 can be measured from urine, non-intrusively and pain-free.

Prior to this study, using a visual analog scale (VAS), senior EPs subjectively reported a greater accumulation of stress, fatigue and more acute feelings of inefficiency during a 24-hour shift (24 hS) compared with a 14-hour night shift (14 hS), with an impact lasting several days [29]. From these observations, we hypothesized that 1) IL-8 levels will be a relevant biomarker of stress and fatigue, sufficiently sensitive to discriminate between two durations of night shifts, 2) IL-8 will be involved in a prolonged response, 3) IL-8 levels in response to shifts will increase with advancing age, 4) IL-8 will identify stressful events surrounding the shifts, and 5) sleep deprivation related to the shifts will exacerbate the IL-8 levels of EPs.

The aim of this study was to assess the impact of 14 hS and 24 hS on urinary IL-8 levels of EPs taking part in the JOBSTRESS trial (Biomarkers of Job Stress: a Randomized Trial among Senior Emergency Physicians) and to investigate the independent associations between stress, fatigue, age, sleep deprivation and workload, and urinary IL-8 levels. We also assessed urinary IL-8 levels three days after shift completion to identify prolonged effects of a preceding shift on the immune function.

## Methods

### Participants

We recruited both men and women to best represent the whole team of the emergency department of the University Hospital of Clermont-Ferrand, France. The protocol was approved by the Regional Ethics Committee of the Clermont-Ferrand University Hospital (CHU), France. The study was explained to EPs who provided their written informed consent. Exclusion criteria were: endocrine disease, pregnancy, recent extraprofessional life stress event (such as death of a near relative, divorce), any current illness, medications used to modulate inflammatory diseases (corticosteroids, anti-inflammatory drugs, immunomodulatory drugs), or any medications with a chronotropic effect taken over the previous six months (beta blockers, diltiazem, verapamil, anxiolytics or antidepressants). We did not exclude non-menopausal women because, to our knowledge, no data have described a link between the menstrual cycle and IL-8. In addition, the few studies on IL-8 and work stress involved mostly women [20], [30], [31], [32], [33], [34].

### Protocol

**Figure 1:**
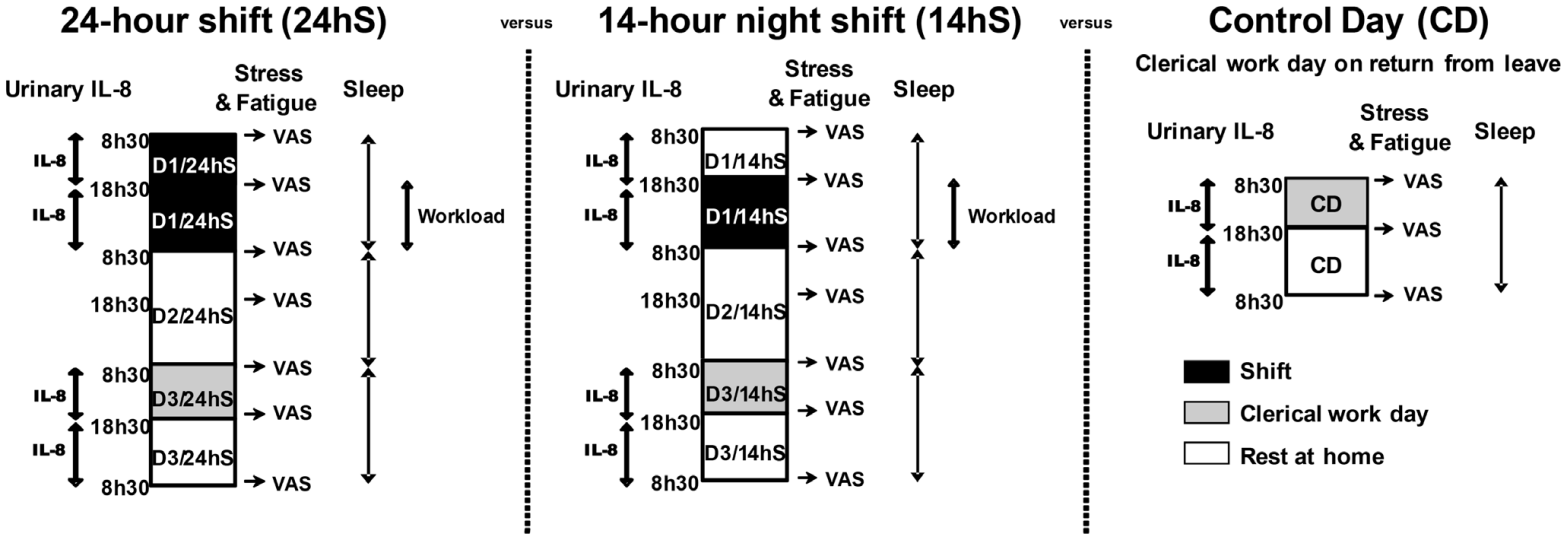
Experimental protocol: three days of follow-up after each type of shift compare with a control day. IL-8: Interleukine-8. VAS: visual analog scale.

For EP, two types of emergency shifts were compared (day 1; D1): the first a 24 hS lasting from 8.30 to 8.30 the following morning (24 hS) and the second a 14 hS from 18.30 to 8.30 (14 hS). Prior to a 14 hS, EPs are at home from 8.30 until 18.30 (Figure 1). To respect their usual routine they were given no particular instructions and were free to do physical activity, take a nap, or other domestic/leisure tasks before commencing the shift. Each shift was followed by a rest day (day 2; D2), as required by French legislation after night work, and by a day of clerical work (day 3; D3). The clerical work day, during which the physicians had no contact with patients, refers to a variety of medical office and administrative support duties including coding recent medical procedures, reading medical images, interpreting patient’s blood sample results, improving skills of students with full-body mannequins that can simulate real-life medical conditions or emergencies, preparing lessons to teach medical students, drafting manuscripts. The clerical work day was added to EPs’ schedule to avoid bias and permit comparisons only of the effects of 24 hS versus 14 hS on IL-8. Finally, each shift was compared with a control day (CD). The control day was standardized to be a clerical work day (8.30–18.30) after returning from at least 8 days of holiday, without jet lag. On this day, they were asked to avoid alcohol and caffeinated beverages and to abstain from heavy physical activity.

All shifts were completed in the medical emergency unit. The study design was observational and based on the existing work schedule. The physicians did not work the day before both shifts.

### Shift Randomization

Latin squares were used to randomize the pattern of shifts and the control day (24 hS then 14 hS then control day or any other combination).

### Assessment of Interleukine-8

Independent of the shift, the EPs were instructed to collect all their urine in separate bottles from 8.30 to 18.30 and 18.30 to 8.30. This was completed during D1 (work), D3 (clerical day) and the control day (CD) (Figure 1). Once retrieved, urine volume was measured and a 2 mL sample was extracted and stored at −80°C for subsequent analysis.

Samples were analyzed blinded to the type of shifts. The same researcher processed all samples using enzyme-linked immunosorbent assay kits (AbCys SA, Paris, France) for measuring IL-8 levels, according to the manufacturer’s instructions. Sensitivity, intra- and inter-assay coefficients of variation were 29 pg/ml, 3.1% and 9.7%, respectively. For all assays, absorbance was measured on a spectrophotometer using a 450 nm as wave length (ELx808™, Bio-tek®, Vermont, USA).

### Assessment of Stress, Mental Fatigue and Physical Fatigue

The psychological consequences of shifts were assessed in terms of perceived stress using a visual analog scale (VAS) [35], [36]. This test assesses the perceived stress level of individuals at work, at home and in daily life on a horizontal, non-calibrated line of 100 mm, ranging from very low (0) to very high (100). We used the same VAS to measure mental and physical fatigue. These measurements occurred at 8.30 and at 18.30 on each day of the protocol. We have previously published results from perceived stress, mental and physical fatigue using VAS [29].

### Workload

The workload during each shift was estimated by: the total number of entries (presentations), the number of admissions, the number of outpatients (collected by computer), and the number of life-and-death emergencies (reported by the EP). When we compared the nights (18 h30 to 8 h30) of both shifts, we limited analyses to workloads the shared times (Figure 1).

### Sleep

Sleep *duration*, including naps, was assessed by questionnaire (bed time - wake time) on the day prior to the shifts, during the three-day tracking of each shift and on the control day.

*Quality* of each period of sleep, including naps, were assessed using the same VAS ranging from very good (0) to very poor (100).

### Statistics

The main judgment criterion was IL-8 levels. Previous data [21] showed that a percent difference in IL-8 levels of approximately 20±20% was required to differentiate between the 24 hS and the 14 hS. Using this difference as the main outcome, we calculated that a sample size of 10 participants allowed a statistical power greater than 80% with an alpha level less than 5%.

Statistical procedures were performed with SPSS Advanced Statistics software version 20 (SPSS Inc., Chicago, IL). The Gaussian distribution for each parameter was assessed by a Shapiro-Wilk test.

Mean levels of IL-8 were compared in the three conditions (24 hS, 14 hS and control day) using Wilcoxon matched-pairs (signed rank) tests, and the independent associations of fatigue, stress, and sleep deprivation with IL-8 levels were further assessed in a multivariable generalized estimating equations model controlling for age, gender and body mass index, and accounting for variation in correlation between repeated measures. The matrix of correlation between the parameters measured was determined using a non-parametric Spearman test. Significance was set at the p<.05 level.

## Results

### Characteristic of the Study Sample

We recruited 19 participants (12 women and 7 men). We obtained full data for 17 physicians out of 19. Two were unable to complete the protocol: one suffered from a stress fracture, while the other was not willing to work a shift of 24 consecutive hours. IL-8 levels were not linked to potential confounding factors such as body mass index, personal status, coffee, smoking, alcohol or regular physical activity. The descriptive characteristics of the EPs are displayed in table 1.

**Table 1:** Descriptive characteristics of emergency physicians.

	All emergency senior physicians	Women	Men
	(n = 17)	(n = 11)	(n = 6)
Age (years)	39.1±6.9	38.8±6.4	39.2±7.0
Body Mass Index (kg/m^2^)	22.8±3.1	22.8±3.7	22.8±1.8
Emergency work experience (years):			
In this emergency unit	6.3±4.8	5.8±4.5	7.4±5.4
In another emergency unit	3.8±5.4	4.1±5.6	2.9±5.2
Number of other medical specialty – in addition to emergency physician	3.9±1.5	3.9±1.4	4.0±2.0
Personal status:			
Married – number (%)	11 (65%)	7 (64%)	4 (67%)
De facto – number (%)	4 (24%)	2 (18%)	2 (33%)
Single – number (%)	2 (12%)	2 (18%)	0 (0%)
Children – mean±SD	1.6±1.5	1.5±1.4	1.7±1.9
Smokers:			
Number of smokers – number (%)	5 (29%)	3 (27%)	2 (33%)
Number of cigarettes/day in a:			
24-hour shift – mean±SD	10.4±4.5	7.3±2.3	15.0±0.0
14-hour shift – mean±SD	5.6±1.8	4.3±0.6	7.5±0.7
control day – mean±SD	4.4±3.0	2.3±1.5	7.5±0.7
Coffee:			
Number of physicians drinking coffee – number (%)	17 (100%)	11 (100%)	6 (100%)
Number of cup/day in a :			
24-hour shift – mean±SD	5.2±2.4	5.5±2.1	4.8±3.1
14-hour shift – mean±SD	3.5±2.0	3.6±2.2	2.6±1.7
control day – mean±SD	4.8±3.1	3.3±1.9	2.5±1.4
Regular physical activity:			
Number of practising – number (%)	13 (76%)	9 (82%)	4 (67%)
Hours per week – mean±SD	4.3±2.4	4.3±2.3	4.3±2.3
Treatment: number of physicians taking a treatment (%) and type of treatment	0	4 (33%) took oral contraceptive pills	0
Alcohol (>3 drink/day for men and >2 drink/day for women)	0	0	0

### Interleukine-8

The circadian level of IL-8 showed no difference between day and night. Therefore, mean levels of different shifts (i.e., 14 hS vs. control day, or 24 hS vs. control day) were compared irrespective of the time of the shift.

**Figure 2:**
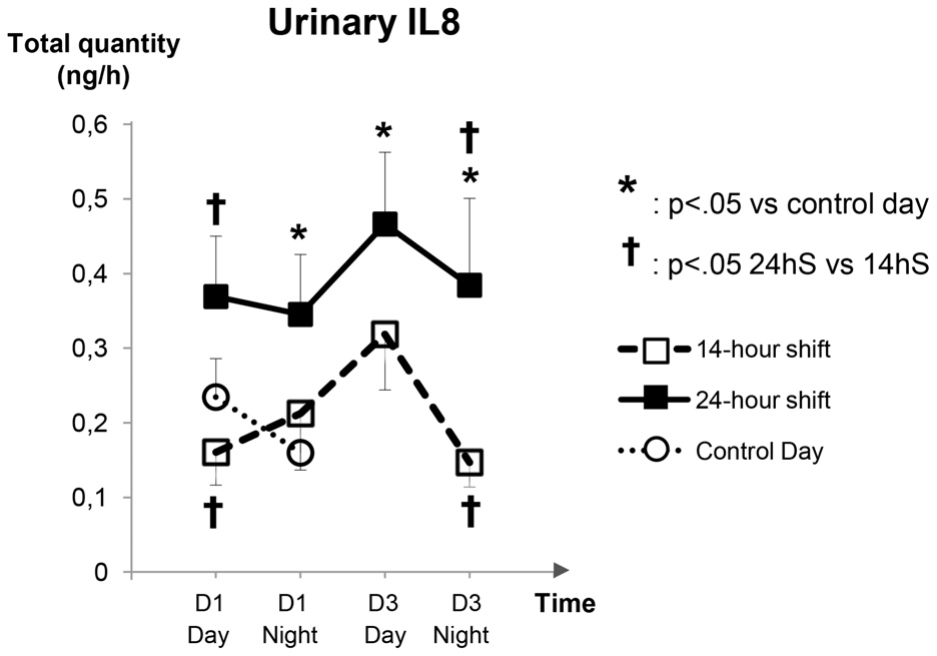
Evolution of urinary IL-8 (mean±SE) during the shifts (Day 1) and on the clerical work day (D3) and during the control day.

Mean IL-8 levels (D1 day+D1 night+D3 day+D3 night) following a 14 hS were similar to those following the control day (p = .7). However, these levels were significantly higher following 24 hS than both the control day (p = .007) and the 14 hS (p = .015). More detailed comparisons of night and day IL-8 levels are presented in figure 2.

### Stress, Fatigue and Sleep

Compared with the control day, mean scores of self-reported stress, mental and physical fatigue, were significantly higher following both the 14 hS and the 24 hS, as previously reported [29].

**Figure 3:**
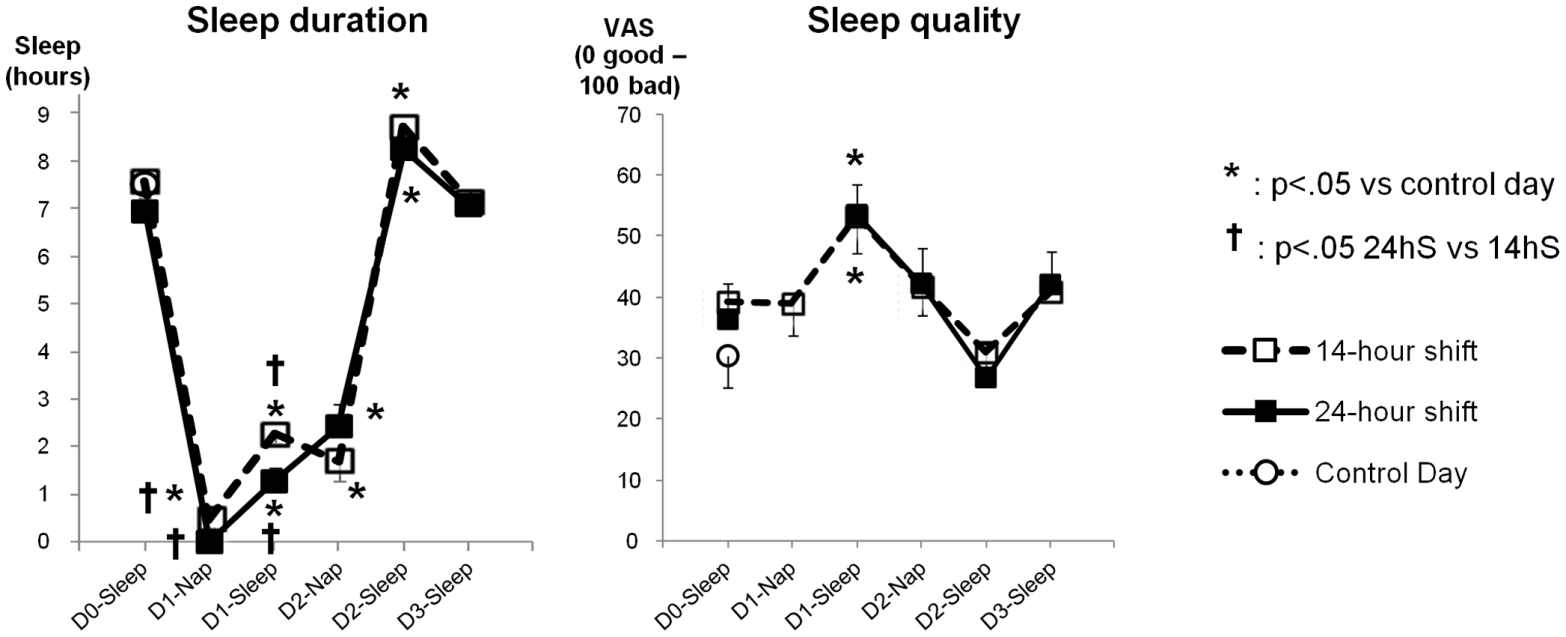
Evolution of sleep duration and sleep quality (mean ± SE) before the shifts (Day 0), during the shifts (D1), the rest day at home (D2), the clerical work day (D3) and during the control day. VAS = visual analog scale.

Self-reported sleep *duration* determined by questionnaires was lower during shifts (24 hS or 14 hS) than on any other day (day before, day following shift, the second day after the shift, and the control day), and was lower during 24 hS than during 14 hS (Figure 3).

*Quality* of sleep was poorest during shifts than any other day, and was also poorer during 24 hS than during 14 hS (Figure 3).

### Correlations

#### Workload

The number of life-and-death emergencies for both shifts was positively correlated with the global amount of IL-8 (D1+D3) (r = .446 p = .017). Furthermore, no associations were found between IL-8 levels and the number of entries, admissions and outpatients.

#### Sleep

The sleep duration *before* the 24 hS was inversely linked to the global amount of IL-8 (r = -.627 p = .007). A *poor quality of sleep* during the shifts was correlated with the global amount of IL-8 (r = .452 p = .031). Conversely, elevated IL-8 levels were associated with a decreased sleep duration the *subsequent* nights.

### Multivariable Analysis

The mean changes in IL-8 levels over time and the impact of covariates on these changes while accounting for variation in the correlation between repeated measures were evaluated using a generalized estimating equations model (Table 2). After controlling for the variables listed in Table 2, engaging in a 24 hS, self-reported stress, and age were the only factors that were independently associated with higher IL-8 levels. A 24 hS significantly increased IL-8 levels by 1.9 ng (p = .007). Similarly, for every unit increase in self-reported stress, there was a 0.11 ng increase in IL-8 levels (p = .003); and for every one year advance in age of physicians, IL-8 levels also increased by 0.11 ng (p = .018). Gender, BMI, mental and physical fatigue, sleep, workload, and engaging in a 14 hS had no independent effects on the IL-8 levels.

**Table 2:** IL-8 levels: a multivariable generalized estimating equations regression.

Covariates	Coefficient (95%CI)	p-value
Age (years), continuous	0.11 (0.02–0.19)	**0.018**
Male gender, (female as reference)	0.81 (−0.33–1.96)	0.164
Body mass index (kg/m^2^),continuous	0.08 (−0.08–0.24)	0.314
14-hour shift!	−0.44 (−1.82–0.93)	0.529
24-hour shift!	1.88 (0.51–3.26)	**0.007**
Mental fatigue, continuous	0.09 (0.00–0.19)	0.058
Physical fatigue, continuous	0.02 (−0.06–0.09)	0.666
Stress, continuous	0.11 (0.04–0.18)	**0.003**
Sleep deprivation!!, continuous	0.01 (−0.03–0.05)	0.535

! Compared with the control shift.

!! Similar results were found when the variables measuring sleep (quantity or quality) were adjusted for.

The model also adjusted for life-and-death emergencies (with no significant associations observed).

## Discussion

The major findings of this study are that urinary IL-8 levels almost doubled during and after a 24 hS compared with a control day or a 14 hS. Furthermore, IL-8 levels failed to return to control values at the end of the third day after the shift, despite a rest day following the 24 hS. This effect was not observed after a 14 hS. In the multivariable model, older age, stressful work conditions and long shifts (i.e. 24-hour) significantly and independently increased the levels of IL-8. Moreover, IL-8 levels were positively correlated with the number of life-and-death emergencies. Finally, sleep deprivation and poor quality of sleep related to the shifts exacerbated IL-8 levels during and after the shifts. All these results supported the hypothesis that IL-8 could be a biomarker of stress, but only under intensive and prolonged demands.

### IL-8 during Shifts: 24 hS vs. 14 hS

We previously reported that 24 hS were perceived to be more tiring and stressful than 14 hS [29]. Here, biological markers also showed that 24 hS is linked to higher IL-8 levels than 14 hS and the control day. Moreover, stressful work conditions significantly and independently increased the levels of IL-8. The results were consistent with earlier findings showing that urinary IL-8 levels of females were correlated to their stress levels [24] and that acute care department nurses, particularly at-risk of a high level of occupational stress [37], had significantly higher secretion levels of IL-8 than the chronic care department nurses [21]. Mood changes, irritability, feelings of stress and fatigue were linked with IL-8 levels in the plasma of healthy subjects [20], [38]. Shift work related to emergency departments, is associated with a high level of negative mood [9], [13], [39]. Therefore, the IL-8 expressions of EPs during their shifts could reflect these negative symptoms. Other studies have compared work during day and night [30], [33] but none to date has compared two types of night work. Prolonged exposure to shift work in EPs is associated with higher rates of substance abuse, depression, suicide, sleep disorders, and burnout from a psychological perspective [9], [13], [39]. From a general health perspective, it is also linked with inflammatory health risks, which have serious implications for cardiovascular disease [10], [13], [40], [41]. Our results suggests that 24 hS may be more detrimental to health that 14 hS. This should be considered in developing shift work rules to prevent future health problems in EPs.

### IL-8 Following Shifts

To our knowledge, there is no publication tracking IL-8 secretion the days *after* night work, namely in an emergency department. Specifically, IL-8 levels remained higher on the third day after the 24 hS than the control day. This was not observed for the 14 hS. The results supported previous findings reporting sustained modifications to heart rate variability at least three days after a 24 hS [29]. Cytokines have also been reported to require as many as five days to return to basal level following an exercise-related stress [22]. Interestingly in our study, physical and mental fatigue remained higher after 24 hS than on the control day. The discrepancy on IL-8 levels between the following days of the two shifts, suggests that IL-8 levels may be not only an appropriate chronic biomarker for evaluating the duration of stress effects on humans but also a sensitive marker for discriminating the level of chronic stress and/or the ability to recover after stress among populations subjected to intense demands. We also suggest that studies on biomarkers of stress should follow the parameters several days after the stress event.

### An Alteration of the Circadian Rhythm of IL-8?

The immune system shows recurring rhythmic variations [42], [43]. The circadian rhythm of IL-8 demonstrates lowest levels during the day from 9.00 to 17.00 and peaks nocturnally from 18.00 to 5.00 [43], which closely represents the period of collection in the present study. The lack of IL-8 circadian rhythm on the control day may suggest a chronic deregulation.

### IL-8 Response to 24 hS Increases with Advancing Age

Cytokine production is increased with age [44], [45], [46]. This low grade inflammation accompanying aging is also responsible for many of the pathologies developing in the elderly [47]. However, in addition to increased secretions with older age, these cytokines are secreted for longer durations while responding to multiple stimuli [23], [47]. Our study is the first to report older age plays is associated with a prolonged immune response during a 24 hS. This may explain the premature departure of EPs following middle age. This also raises more questions regarding the impact of repeated 24 hS on health throughout their career.

### IL-8 and Life-and-death Emergencies

Although only based on correlations, we failed to demonstrate a link between IL-8 levels and general workload indicators including the number of entries, admissions or outpatients. However, life-and-death emergencies may appear as a potential indicator of stress for EPs, which supports our previous findings [27]. Our results may possibly link to the increased morbidity observed in subsequent patients when cardiac surgeons are confronted with a patient’s death in emergency surgery [48]. The potential for residual effects after life-and-death events in emergency departments need to be investigated.

### IL-8 is a Marker of Stress and Sleep Deprivation

Again within the limited results of correlations, we report the link between sleep deprivation and IL-8 levels. These findings support previous studies demonstrating that sleep deprivation also exacerbates other pro-inflammatory cytokines secretion [25], [26], [27], [28] or high sensitivity CRP [49]. Thus, we speculate that elevated IL-8 levels observed during and following the shifts were linked to sleep deprivation that were exacerbated by the additional work stressors such as life-and-death emergencies. Moreover, the duration of stress could play a major role because we observed an increase in IL-8 levels only after 24 hours of uninterrupted work, which is a common occupational exposure for EPs in some hospitals. Poor sleep can influence inflammatory cytokines and when elevated, these same cytokines can negatively impact on sleep [50]. This is supported by our results showing that elevated IL-8 levels during shifts were associated with poor sleeping patterns the days following shifts. Chronic sleep deprivation increases inflammation and perturbs the circadian clock which in turn, increases susceptibility to diseases such as diabetes, obesity, cancer [51], and cardiovascular diseases [49]. Many EPs are rostered to a 24 hS at least once a week throughout their working lives and our study profiles one shift having prolonged effects on the immune system. Therefore, we propose that more EPs should share the night shifts which may lowered their stress and permit more sleep. This could be investigated in a future research.

### Strengths and Limitations

The strengths of the present study include: randomized order of the shifts, a run-in design, participants being their own control, adequate statistical power to support the primary hypothesis, factors modifying IL-8 were identified, workload was assessed objectively and subjectively, and the innovation in being the first study to compare two types of night work and following participants into the recovery period.

There are a number of limitations to this study: external validity may not extend to emergency departments that have less intensive workloads (48 000 entries per year, 30% hospitalized, for 16 full-time equivalent EPs, 52 nurses, with 14 available beds), and may also lack some external validity because of the data profiles only one emergency department. Duties and shifts of emergency physicians may vary internationally but systemic responses to life-and-death emergencies remain under researched. Our results provide opportunities to generate a number of hypotheses for future research.

### Conclusions

In conclusion, urinary IL-8 is a convenient and non invasive strong biomarker of stress, both acutely and over time. IL-8 was highest during 24-hour shifts. Moreover, the 24-hour shifts were also involved in a prolonged response of the immune system, with increased IL-8 levels at least three days after the shift. Finally, IL-8 secretion in response to 24-hour shifts also increased with advancing age of physicians. Since elevated IL-8 levels are associated with cardiovascular disease and negative psychological consequences, we suggest that emergency physicians limit their exposure to 24 hS, especially with older age.
